# Design and optimization of cranberry extract loaded bile salt augmented liposomes for targeting of MCP-1/STAT3/VEGF signaling pathway in DMN-intoxicated liver in rats

**DOI:** 10.1080/10717544.2022.2032875

**Published:** 2022-01-31

**Authors:** Sara M. Soliman, Shaimaa Mosallam, Mohamed A. Mamdouh, Mohammed Abdalla Hussein, Shady M. Abd El-Halim

**Affiliations:** a Department of Pharmaceutics and Industrial Pharmacy, Faculty of Pharmacy, October 6 University, 6^th^ of October City, Giza, 12585, Egypt; b Biochemistry Department, Faculty of Applied Medical Sciences, October 6 University, 6^th^ of October City, Giza, 12585, Egypt

**Keywords:** Cranberry extract, antioxidant, bile salt augmented liposomes, Dimethylnitrosamine, liver injury

## Abstract

Cranberry extract (CBE) is a major source of the antioxidant polyphenolics but suffers from limited bioavailability. The goal of this research was to encapsulate the nutraceutical (CBE), into bile salt augmented liposomes (BSALs) as a promising oral delivery system to potentiate its hepatoprotective impact against dimethylnitrosamine (DMN) induced liver injury in rats. The inclusion of bile salt in the liposomal structure can enhance their stability within the gastrointestinal tract and promote CBE permeability. CBE loaded BSALs formulations were fabricated utilizing a (2^3^) factorial design to explore the impact of phospholipid type (X_1_), phospholipid amount (X_2_), and sodium glycocholate (SGC) amount (X_3_) on BSALs properties, namely; entrapment efficiency percent, (EE%); vesicle size, (VS); polydispersity index; (PDI); zeta potential, (ZP); and release efficiency percent, (RE%). The optimum formulation (F1) exhibited spherical vesicles with EE% of 71.27 ± 0.32%, VS; 148.60 ± 6.46 nm, PDI; 0.38 ± 0.02, ZP; −18.27 ± 0.67 mV and RE%; 61.96 ± 1.07%. Compared to CBE solution, F1 had attenuated DMN-induced hepatic injury, as evidenced by the significant decrease in serum level of ALT, AST, ALP, MDA, and elevation of GSH level, as well as SOD and GPX activities. Furthermore, F1 exhibited an anti-inflammatory character by suppressing TNF-α, MCP-1, and IL-6, as well as downregulation of VEGF-C, STAT-3, and IFN-γ mRNA levels. This study verified that when CBE was integrated into BSALs, F1, its hepatoprotective effect was significantly potentiated to protect the liver against DMN-induced damage. Therefore, F1 could be deliberated as an antioxidant, antiproliferative, and antifibrotic therapy to slow down the progression of hepatic damage.

## Introduction

The liver is responsible for a wide range of essential functions in the body. Many hepatotoxins cause hepatic dysfunction (Rasheed et al., 2021), including industrial chemicals, plant poisons, herbal treatments, nutritional supplements, and medicines. Liver fibrosis is a consequence of persistent liver injury that exceeds the liver’s ability to regenerate. Portal hypertension, cirrhosis, cancer, and liver failure are major complications of progressive liver fibrosis (Bataller & Brenner, 2005). As yet, no particular and effective antifibrotic treatment has been documented (Lee et al., 2021).

Many phytochemicals found in fruits, whole grains, and vegetables are recognized to be powerful antioxidants (Liu, 2013). They have garnered a lot of attention in the management of hepatic injury due to their relative safety compared to traditional synthetic agents (Latief & Ahmad, 2018). As a result, the introduction of new functional foods and nutraceuticals produced from plants, which are already well-known as edible sources with high antioxidant content, has been widely investigated in recent years (Călinoiu et al., 2019). Among these plants, Vaccinium species are frequently mentioned for their range of phenolic compounds (Bujor et al., 2018), particulary cranberry (Vaccinium macrocarpon Aiton) is contributed due to its widespread consumption rate in a variety of forms.

Interestingly, cranberry extract (CBE) is regarded to be a beneficial nutraceutical source of phytochemicals, primarily polyphenols enriched with flavonols (Blumberg et al., 2013), which have been evinced to have an antioxidant effect via neutralizing the reactive oxygen species (ROS), chelating metal ions, upregulating, and activating antioxidant enzymes and thereby interfering with the cell signaling pathways (Caldas et al., 2018). Despite its therapeutic efficiency, CBE is reported to undergo fast removal from plasma as a result of rapid metabolism and renal clearance, as well as having a low bioavailability due to its restricted intestinal absorption (Caldas et al., 2018). Hence, the encapsulation of CBE in an advanced nanosystem is expected to achieve superior results by improving its bioavailability and therapeutic efficiency against liver damage.

Liposomes are phospholipid vesicles that have long been used as the preferred delivery system for hydrophilic and hydrohpobic drugs (Elnaggar, 2015) due to biocompatibility, excellent entrapment capacity and safety. Nevertheless, phospholipid vesicles are susceptible to digestion in the gastrointestinal tract by gastric acid and pancreatic secretions and have poor permeability through the intestinal membrane because of liposomes’ relatively large size following oral administration (Hu et al., 2013, Freag et al., 2013). Bile salts have been exploited to promote the absorption, integrity and stability of liposomes (He et al., 2019). Therefore, the incorporation of bile salts into the lipid bilayer of liposomes contributes to the protection of liposomes from the detrimental effects of physiological secretions inside the gastrointestinal tract. Moreover, the presence of bile salts in the liposomal structure may have a membrane-destabilizing effect when they come into contact with the intestinal epithelia, facilitating vesicles’ internalization (Niu et al., 2012, Hu et al., 2013).

Different types of bile salts have been utilized in oral drug delivery, such as sodium deoxycholate, sodium taurocholate, and sodium glycocholate (SGC) (Hu et al., 2013). Among those, SGC is the most commonly utilized because of its lower toxicity, its capability to enhance drug permeability, and its greater ability to inhibit the proteolytic enzymes in the gastrointestinal tract, protecting the liposomes against digestion (Saifi et al., 2020). As reported by (Niu et al., 2012), SGC containing liposomes provided better protection and greater oral bioavailability for insulin compared to sodium taurocholate or sodium deoxycholate, or the traditional liposomes.

Epikuron 100 and Epikuron 200 are phospholipids in nature and are used in the fabrication of lipid-based drug delivery systems to enhance the delivery of our drug candidate, CBE. They are derived from soybean lecithin, where they differ in the concentration of phosphatidylcholine (20% and 92% phosphatidylcholine for Epikuron 100 and Epikuron 200, respectively).

To date, no research has explored the encapsulation of CBE into nanosystems to improve its oral bioavailability and hence its hepatoprotective impact. Accordingly, this study was conducted to fabricate CBE loaded BSALs formulations and assess the impact of various formulation variables on BSALs characteristics and figure out the best formulation according to the optimized criteria through the use of Design-Expert® software. Additionally, the potential antifibrotic impact of CBE loaded BSALs in dimethylnitrosamine (DMN)-induced chronic liver injury in rats was investigated. Moreover, our approach was clarified by investigating various oxidative, inflammatory markers, and fibrotic mediators.

## Methods

### Formulation of CBE loaded BSALs

The thin film hydration tactic was utilized to fabricate CBE loaded BSALs (Albash et al., 2019a). They were formulated by varying the phospholipid type and the amounts of both the phospholipid and SGC. Initially, the phospholipid was completely dissolved in a (3:1 v/v) mixture of methylene chloride: methanol in a 250 ml flask with round bottom. Then, the organic phase was evaporated under vacuum at 60 °C utilizing a rotary evaporator. The film was then hydrated with 10 ml of deionized water, incorporating CBE (60 mg/1 ml of each formulation) and SGC, for one hour at the same temperature, which is above the phase transition temperature of lipid, followed by probe sonication for 1 min (3 sec on, 2 sec off and amplitude 40%) to diminish vesicle size and kept at 4 °C to attain maturation.

## In vitro *characterization and optimization of CBE loaded BSALs*

### CBE loaded BSALs entrapment efficiency percent (EE%)

The percentage of CBE EE in BSALs was estimated by indirect determination of the unentrapped CBE applying the ultrafiltration-centrifugation method (Vitorino et al., 2011). Briefly, one ml of each formulation was placed into the upper chamber of the centrifugal filtration unit, which was subsequently centrifuged at 10000 rpm for one hour at 4 °C. The unentrapped CBE was determined in the outer chamber of the centrifugal unit using UV/VIS spectrophotometer at λ_max_ 280 nm. CBE EE% was estimated by applying the equation below:

(1) $$\mathrm{EE}\;(\%)=\frac{\text{Initial CBE amount }-\;\text{Free CBE}}{\text{Initial CBE amount}}\;\times 100$$

### CBE loaded BSALs vesicle size (VS), polydispersity index (PDI), and zeta potential (ZP)

The mean VS, PDI, and ZP of the various fabricated CBE loaded BSALs were examined using Malvern Zetasizer (Abd El-Halim et al., 2021).

### CBE loaded BSALs release efficiency percent (RE%)

The measurement of RE% of CBE from the fabricated BSALs formulae was carried out using a dialysis method (Khan et al., 2015). One ml of each formulation, equivalent to 60 mg CBE, was placed in the presoaked cellulose dialysis bag. The bag was then clamped and placed in a beaker containing 20 ml distilled water as a receptor compartment. The system was kept at 37 °C while being continuously stirred using a magnetic stirrer at (100 rpm). Aliquots of (1 ml), at predefined time intervals up to 24 hours, were collected from the receptor compartment and replenished with an equal volume of fresh medium. RE% was quantified by employing the following equation (Sharma et al., 2015).

(2) $$RE\%=\frac{\int_{t_{1}}^{t_{2}}y.dt}{y_{100}\;\times (t_{2}-t_{1})}\;\times 100$$

where y is the % of the product released, RE% is the area under the release curve from time points t_1_ to t_2_ represented as a percentage of the curve at maximum release y_100_ at the end of the release period.

### Experimental design construction

Initial trials were conducted (data is not displayed) to determine the possible arrays of the independent variables. 2^3^ factorial design was employed using Design-Expert^®^ software (Version 7, Stat-Ease Inc., USA) to investigate the impact of various factors in fabricating CBE loaded BSALs. The effects of three independent variables, at two levels each, were estimated, namely; phospholipid type (X_1_), phospholipid amount (X_2_), and SGC amount (X_3_). The EE% (Y_1_), VS (Y_2_), PDI (Y_3_), ZP (Y_4_), and RE% (Y_5_) were nominated as the dependent variables (Table 1).

**Table 1.** 2^3^ full factorial design utilized for optimization of BSALs formulations.

Factors (independent variables)	Levels	
X_1_: Phospholipid type	Epikuron 100	Epikuron 200
X_2_: Phospholipid amount (mg)	600	900
X_3_: SGC amount (mg)	15	30
**Responses (dependent variables)**	**Desirability constraints**	
Y_1_: EE%	Maximize	
Y_2_: VS (nm)	Minimize	
Y_3_: PDI	In the range	
Y_4_: ZP (mV)	In the range	
Y_5_: RE%	Minimize	

BSALs: bile salt augmented liposomes; SGC: sodium glycocholate; EE%: entrapment efficiency percent; VS: vesicle size; PDI: polydispersity index; ZP: zeta potential; RE%: release efficiency percent.

### Optimization of CBE loaded BSALs

The desirability was optimized according to the criteria of the highest EE%, smallest VS, and RE% while maintaining the values of PDI and ZP in their range to attain the optimum formula. A desirability value close to (1) was picked. To demonstrate the effectiveness of this design, the selected formula was prepared, assessed, and compared to the predicted values.

### Characterization of the optimum CBE loaded BSALs

#### Transmission electron microscopy (TEM)

TEM was employed to investigate the morphological appearance of the optimum CBE loaded BSALs. The stained formulation was deposited on a carbon grid with a copper coat and permitted to dry until TEM analysis (Abd El-Halim et al., 2020).

#### The optimum CBE loaded BSALs stability

The optimum CBE loaded BSALs formulation was kept at 4–8 °C for 45 days (Abd-Elsalam et al., 2018). The stability was assessed by checking the changes in appearance, EE%, VS, PDI, ZP, and RE% of the optimum formula after storage. The observed results were statistically analyzed using Student’s *t*-test by SPSS^®^ 25.0 software.

#### Determination of the optimum CBE loaded BSALs cytotoxicity (IC_50_) on liver carcinoma (Hep-G2) cell line

The effect of the optimum CBE loaded BSALs on the viability of Hep-G2 cell lines was assessed by applying MTT assay. A 24-well plate was loaded with cells at a density of 1.0 × 10^6^ cells/well. For 24 h, the Hep-G2 cells were subjected to the optimum CBE loaded BSALs at concentrations of 31.5, 62.5, 125, 250, 500, and 1000 μg/ml. BSALs free cells were employed as controls. Following exposure, cells were incubated with MTT (20 μL/well) for 4 hours, so that, in living cells, mitochondrial dehydrogenases transform the dissolved yellowish MTT into formazan crystals (water insoluble) that could be dissolved in DMSO. Centrifugation was used to separate the medium from the suspension culture, and 200 μL of DMSO was introduced to solubilize the produced crystals where the optical density was gauged using a microplate reader at 570 nm (Biotek, USA).

## In-vivo *studies of the optimum CBE loaded BSALs*

### Animals

Male Wistar rats (250 g) were purchased from the National Cancer Institute’s animal house, Cairo University, Giza, Egypt. Rats were fed with standard diet *ad-libitum* and water, observed daily, and kept in polypropylene cages under constant environmental conditions (12:12 h light: dark cycle at around 22 °C) throughout the experimental work. All experiments were carried out in accordance with the standards of the Ethics Committee of the Faculty of Applied Health Sciences Technology, October 6 University, Egypt (Registration No. 20210115).

### Determination of LD_50_ of the optimum CBE loaded BSALs

The LD50 was estimated in groups of ten animals to define the oral dose range of the optimum CBE loaded BSALs that leads to the death of animals from zero to 100%. The optimum formulation was administered orally at different doses of 350, 500, 800, 1200, 1600, and 1800 mg/kg. Animals were individually monitored every hour for the first day and daily over the next five days following the administration of the tested formulation. Saganuwan’s method (Saganuwan, 2011) was used to calculate the LD_50_ using the following formula:

(3) LD50 = Dm−[∑ (Z.d)n ]

where Dm = the toxic dose that causes mortality in all animals, Z = The average number of dead animals between two succeeding groups, d = the interval between every two successive doses, ∑ (Z.d) = the sum of (Z x d), and n = the total number of animals used in each group.

### Induction of liver injury with DMN

The rats were randomly divided into six groups (ten rats each). Group I (served as the vehicle-treated normal control) received intraperitoneal normal saline solution once a day, 3 days a week, for a total of 11 weeks. Group II (positive control; DMN) rats received a daily intraperitoneal injection of 2 ml/kg of 0.5% (v/v) DMN for three sequential days/week, for four weeks to induce the hepatic injury (Xiang et al., 2017). In group III (CBE), the rats were administered CBE dissolved in deionized water (75 mg/kg/day; p.o.) for 7 weeks. In group IV (CBE + DMN), the rats were administered CBE dissolved in deionized water (75 mg/kg/day; p.o) for 7 weeks then received 0.5% (v/v) of DMN (2 ml/kg/day; i.p) for three sequential days/week, for four weeks. Group V (F1), the rats were administered the optimized formulation in a dose of 1/20 LD50 (53.13 mg/kg/day; p.o) for 7 weeks. Group VI (F1 + DMN), the rats were administered the optimized formulation F1 (53.13 mg/kg/day,p.o) for 7 weeks then received 0.5% (v/v) of DMN (2 ml/kg/day; i.p) for 3 sequential days/week, for 4 weeks .

### Blood and tissue sampling

Blood samples were taken at the end of the investigational period from overnight fasted rats through a heart puncture following light ether anesthesia. The collected blood samples were permitted to coagulate at 37 °C for 15 min, then centrifuged at 3000 rpm for 15 min. Serum portions were separated and aliquoted to be stored at −20 °C till they were used in the biochemical determination. Aspartate aminotransferase (AST), alanine aminotransferase (ALT), and alkaline phosphatase (ALP) were determined in the stored sera.

Immediately after the animals were sacrificed, livers were dissected from rats of the different groups; quickly excised, rinsed with normal saline and blotted with filter paper. The collected livers were divided into two parts as follows: the first part was placed in ice-cold saline and homogenized to ultimately yield 10% (w/v) whole tissue homogenates. The homogenates were then centrifuged at 3000 rpm at 4 °C for 10 min, and the supernatants were aspirated and used for the analysis of the levels of tumor necrosis factor (TNF-α), Interleukin-6 (IL-6), Monocyte Chemoattractant Protein-1 (MCP-1), malondialdehyde (MDA). The second part of the liver was used for the determination of gene expression levels of Interferon-γ (IFN-γ), Vascular endothelial growth factor C (VEGF-C), and signal transducer and activator of transcription 3 (STAT3) by real-time PCR.

### Determination of liver function biomarkers

Siemens Dimension Vista system has been used to assess the activity of hepatic function biomarkers. AST and ALT levels were determined according to the method introduced by (Bergmeyer et al., 1978). The activities of ALP were evaluated using the technique outlined in (Bowers & McComb, 1966).

### Measurement of oxidative stress (homogenate redox) biomarkers

The levels of lipid peroxidative products were measured colorimetrically in liver tissue homogenates using the thiobarbituric acid test for MDA (Yoshioka et al., 1979) and using commercially available kits (Biodiagnostic, Cairo, Egypt) to evaluate the levels of glutathione (GSH), glutathione peroxidase (Gpx), and superoxide dismutase (SOD), following the instructions provided in the kit manuals, according to the method of (Wang et al., 2020).

### Measurement of inflammatory and proinflammatory factors

Serum levels of MCP-1 (Ogata et al., 1997), TNF-α, and IL-6 (Song et al., 2011) were assessed in units of pg/ml using a commercial ELISA kit obtained from (Quantikine R, Minnesota, USA).

## RNA *extraction and real-time polymerase chain reaction (RT-PCR)*

The liver was rapidly removed when the rats were slaughtered, and a little section was pulverized in liquid nitrogen using RNase-free instrument. Subsequently, the TRIzol reagent was combined with the pulverized samples. The gene expression of IFN-γ, VEGF-C, and STAT3 was then determined using real-time PCR (RT-PCR). All data for the quantification of mRNA were assigned to glyceraldehyde 3-phosphate dehydrogenase (GAPDH). The Sequence Detection Program (PE Biosystems, CA, USA) was used to perform the RT-PCRs in a thermal cycler stage one plus (Applied Biosystems, USA). Table S1 cites the oligonucleotides utilized in these amplifications. The conditions for the thermal cycling of PCR included pre-incubation at 50 °C for 2 minutes, followed by 40 cycles at 95 °C for 15 sec and 60 °C for 1 min.

### Statistical analysis

IBM SPSS program (version 22 Inc., Chicago, USA) was used to do the statistical studies. The disparities between groups were computed using one-way analysis of variance (ANOVA) followed by Duncan’s test post hoc analysis. All values were recorded as Mean ± SD, and differences were regarded as significant at (*p* < .05).

## Results and discussion

### Factorial design outcomes

**Figure 1.**
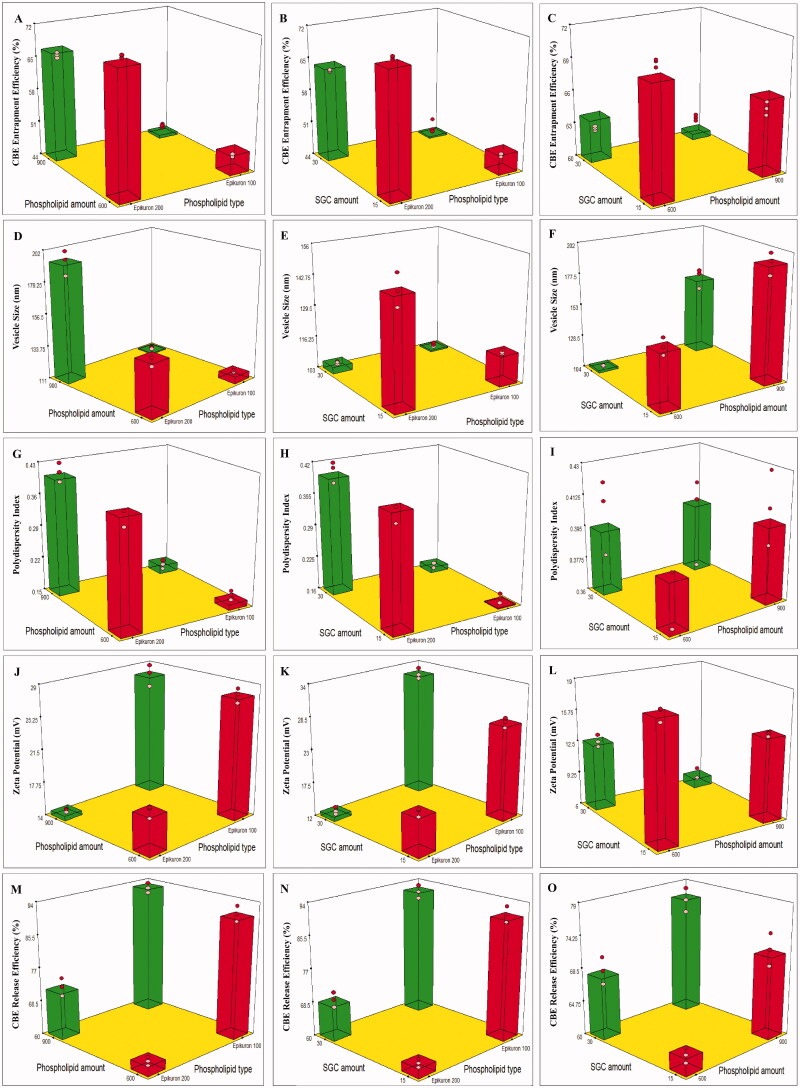
3D plots illustrating the effect of phospholipid type, phospholipid amount and SGC amount on (A–C) % CBE entrapment efficiency, (D–F) vesicle size, (G–I) polydispersity index, (J–L) zeta potential and (M–O) % CBE release efficiency.

Referring to the outcomes of the design analysis, the two-factor interaction (2FI) model has been used, and it was observed that the predicted (R^2^) outcomes were in rational accordance with the adjusted (R^2^) in all responses. A ratio of adequate precision higher than four is desirable, and that was noticed in all the explored responses, as displayed in (Table 2). Figure 1 depicts the effect of different independent variables; phospholipid type (X1), phospholipid amount (X2), and SGC amount (X3) on the EE% (Y1), VS (Y2), PDI (Y3), ZP (Y4), and RE% (Y5) of CBE loaded BSALs.

**Table 2.** A) 2^3^ factorial analysis outcomes of BSALs formulations and B) the observed and predicted outcomes of the optimum CBE loaded BSALs (F1).

A) Responses	R^2^	Adjusted R^2^	Predicted R^2^	Adequate precision	Significant factors
EE%	0.99	0.98	0.97	35.03	X_1_, X_2_, X_3_
VS (nm)	0.98	0.98	0.97	36.15	X_1_, X_2_, X_3_
PDI	0.98	0.98	0.96	24.26	X_1_
ZP (mV)	0.99	0.99	0.98	47.22	X_1_, X_2_, X_3_
RE%	0.98	0.98	0.97	31.07	X_1_, X_2_, X_3_
B) Response	Y_1_ EE%	Y_2_ VS	Y_3_ PDI	Y_4_ ZP	Y_5_ RE
Observed values	71.27	148.60	0.38	−18.27	61.96
Predicted values	70.09	148.27	0.39	−18.29	62.44

CBE: cranberry extract; BSALs: bile salt augmented liposomes; EE%: entrapment efficiency percent; VS: vesicle size; PDI: polydispersity index; ZP: zeta potential; RE%: release efficiency percent.

### The significance of formulation variables on the EE% of CBE loaded BSALs

The capability of the prepared BSALs to encapsulate a high amount of drugs is the major obstacle to promoting oral delivery. The percentage of CBE entrapped within BSALs fluctuated between 39.50 ± 0.87 and 71.27 ± 0.32% (Table 3).

**Table 3.** Evaluation of the different responses of CBE loaded BSAls formulae.

Formulations	X_1_ Phospholipid type	X_2_ Phospholipid amount (mg)	X_3_ SGC amount (mg)	Y_1_ EE%	Y_2_ VS (nm)	Y_3_ PDI	Y_4_ ZP (mV)	Y_5_ RE%
F1	Epikuron 200	600	15	71.27 ± 0.32	148.60 ± 6.46	0.38 ± 0.02	−18.27 ± 0.67	61.96 ± 1.07
F2	Epikuron 200	900	15	65.70 ± 0.60	193.70 ± 8.48	0.41 ± 0.02	−14.53 ± 0.21	72.40 ± 2.33
F3	Epikuron 100	600	15	47.05 ± 0.30	115.57 ± 0.86	0.17 ± 0.01	−27.77 ± 0.81	91.02 ± 1.92
F4	Epikuron 100	900	15	45.80 ± 0.40	111.90 ± 0.52	0.16 ± 0.01	−27.87 ± 1.27	92.60 ± 1.31
F5	Epikuron 200	600	30	62.70 ± 0.20	105.50 ± 0.66	0.40 ± 0.02	−12.77 ± 0.60	69.39 ± 1.95
F6	Epikuron 200	900	30	61.70 ± 0.28	162.97 ± 8.05	0.39 ± 0.03	−6.93 ± 0.67	76.50 ± 1.78
F7	Epikuron 100	600	30	45.57 ± 1.57	104.00 ± 0.95	0.16 ± 0.01	−32.43 ± 0.91	91.65 ± 1.72
F8	Epikuron 100	900	30	39.50 ± 0.87	110.03 ± 0.23	0.17 ± 0.01	−30.60 ± 2.03	93.75 ± 2.00

All of the fabricated BSALs included an equal quantity of CBE (60 mg/1 ml of each formulation). Data are provided as mean ± SD, (*n* = 3). CBE: cranberry extract; BSALs: bile salt augmented liposomes; SGC: sodium glycocholate; EE%: entrapment efficiency percent; VS: vesicle size; PDI: polydispersity index; ZP: zeta potential; RE%: release efficiency percent.

Phospholipid type (X_1_) had a significant influence on EE% (*p* < .0001). BSALs fabricated using Epikuron 200 exhibited higher EE% than those fabricated using Epikuron 100. This could be explained by the difference in phosphatidylcholine content between Epikuron 200 (92%) and Epikuron 100 (20%). The increase in phosphatidylcholine content contributes to superior packing of the lipids resulting in higher CBE loading (Rajendar & Saraswathi, 2014).

Increasing the phospholipid amount (X_2_) resulted in a significant reduction in EE% (*p* < .0001). This could be driven by the assumption that the EE% of hydrophilic compounds depends on the encapsulated fraction of the aqueous phase during vesicles’ formation (Fan et al., 2021), and higher amounts of phospholipids might lead to vesicles’ aggregation and subsequently decrease the number of vesicles and the aqueous core taking part in encapsulation (Soliman et al., 2016). Similar results were observed by (Nandhini & Ilango, 2021) and (Hao & Li, 2011) who reported a decrease in EE% with increasing the total amount of lipids. Similarly, when the amount of SGC (X_3_) increased, the EE% decreased significantly (*p* < .0001). This might be explicable by the fluidizing impact of SGC on the bilayer of the vesicles at high concentrations, which might have allowed the leakage of the encapsulated CBE (Niu et al., 2011).

### The significance of formulation variables on the VS of CBE loaded BSALs

It is well known that the fabrication of nanoscale range systems contributes to improved oral drug delivery. VS of CBE loaded BSALs varied from 104.00 ± 0.95 to 193.70 ± 8.48 nm, as displayed in (Table 3). Phospholipid type (X_1_) had a significant impact on VS (*p* < .0001). BSALs prepared using Epikuron 200 manifested larger VS than those prepared using Epikuron 100, which could be justified by the higher amount of CBE entrapped within the vesicles prepared using Epikuron 200, as formerly stated. The elevation in the quantity of CBE entrapped within the vesicles increases the gap expanding between the bilayers and, thereby, the vesicles are enlarged (Hathout et al., 2007).

Concerning the phospholipid amount (X_2_), a higher amount of phospholipid resulted in increased VS significantly (*p* < .0001). This could be justified by assuming that the existence of high content of phospholipid led to a rise in the amount of lipid in each vesicle, with a consequent increase in the VS (Mosallam et al., 2021a). On the other hand, increasing SGC amount (X_3_) led to a significant lowering of the VS (*p* < .*0001*). This might be justified in terms of the lower amount of CBE entrapped within the vesicles at a higher SGC level, as mentioned earlier (Hathout et al., 2007).

### The significance of formulation variables on the PDI of CBE loaded BSALs

The PDI of all formulae ranged from 0.16 ± 0.01 to 0.41 ± 0.02 (Table 3). Based on the factorial analysis outcome, only phospholipid type (X_1_) had a significant influence on the PDI (*p* < .*0001*). The use of Epikuron 100 resulted in vesicles with lower PDI and better homogeneity, which might be ascribed to the small VS range compared to Epikuron 200 (El Kayal et al., 2020).

### The significance of formulation variables on the ZP of CBE loaded BSALs

As demonstrated in (Table 3), the fabricated CBE loaded BSALs exhibited negative ZP values that ranged from −6.93 ± 0.67 to −32.43 ± 0.91 mV. The negative charge is probably imparted by the phospholipid (Subongkot et al., 2014) and the anionic nature of SGC (Mosallam et al., 2021b). ANOVA analysis revealed that all three independent variables (X_1_, X_2_, and X_3_) had a significant influence on ZP, (*p* < .0001 for X_1_ and X_2_ and *P =* 0.0028 for X_3_). Regarding phospholipid type (X_1_), BSALs prepared using Epikuron 100 manifested higher ZP values than those prepared using Epikuron 200. This might be explained by the larger VS obtained by Epikuron 200, which resulted in a reduction of the surface area carrying charge (El Kayal et al., 2020).

Similarly, increasing the amount of phospholipid (X_2_) caused a significant reduction in ZP values, which might be explained by the larger VS obtained at higher phospholipid levels, as mentioned before. Despite the fact that SGC stabilizes the vesicles by imparting surface charge, increasing SGC amount (X_3_) significantly lowered ZP values, which could be assigned to the collapse in the electrostatic layer and the consequent decrease in ZP above a certain level of SGC amount. Similar outcomes were obtained by (Aziz et al., 2019) while formulating diacerein loaded bilosomes.

### The significance of formulation variables on the RE% of CBE loaded BSALs

**Figure 2.**
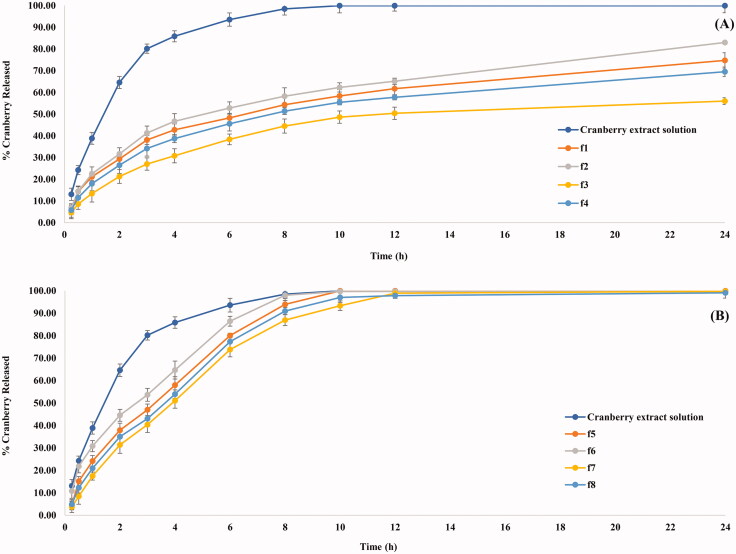
In-vitro CBE release profile from CBE loaded BSALs prepared using (A) low SGC amount and (B) high SGC amount.

Figure 2 shows a sustained release pattern of CBE from the different formulae with RE% ranging from 61.96 ± 1.07 and 93.75± .00% (Table 3). Concerning the phospholipid type (X_1_), BSALs fabricated using Epikuron 200 had a significantly lower RE% compared to Epikuron 100 (*p* < .0001). This might be ascribed to the higher phosphatidylcholine content for Epikuron 200 (92%), which results in firmer lipid membranes with a consequent hindering of the drug release (Aboud et al., 2016, Albash et al., 2019a).

A significant decrease in the RE% (*p* < .*0001*) was observed at lower amounts of phospholipid (X_2_). This could be explained by the higher amount of CBE entrapped within vesicles containing a low amount of phospholipid, which increases the time necessary for CBE diffusion from the vesicles to the external environment. As a result, it provides a sustained release pattern (Waghule et al., 2021). Moreover, increasing SGC amount (X_3_) significantly increased RE% (*P = 0.0004*). This could be explained by the ability of SGC to associate with the vesicles’ bilayers and transform them into mixed micelles, which contributes to an increase in the solubility of CBE and hence increased RE%. These findings were in accordance with the results obtained by (Albash et al., 2019b).

### Determination of the optimum CBE loaded BSALs formulation

The aim of the optimization process was to attain CBE loaded BSALs having the maximum EE%, minimum VS, and RE% while keeping the values of PDI and ZP in range. Accordingly, the optimum formula (F1), which possessed an EE% of 71.27 ± 0.32%, VS of 148.60 ± 6.46 nm, PDI of 0.38 ± 0.02, ZP of −18.27 ± 0.67 mV, and RE% of 61.96 ± 1.07%, was selected with a desirability value (d = 0.829). A high correlation was found among the observed and predicted outcomes of F1 (Table 3). Subsequently, the optimum CBE loaded BSALs formulation (F1) was chosen for additional investigations.

### Characterization of the optimum CBE loaded BSALs

#### Transmission electron microscopy (TEM)

**Figure 3.**
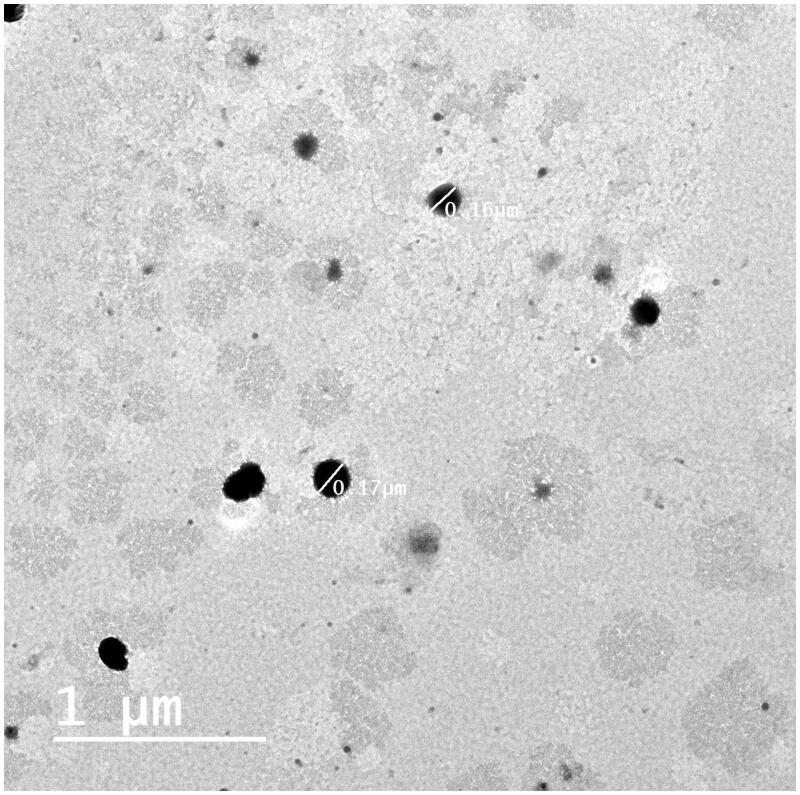
TEM photomicrograph of the optimum CBE loaded BSAL (F1).

The TEM micrograph of the optimum CBE loaded BSALs (F1) revealed non-aggregated spherical vesicles with a rather uniform size distribution, (Figure 3). The observed VS was commensurate with the results obtained by Zetasizer.

#### The optimum CBE loaded BSALs stability

The statistical analysis exposed that there was a non-significant variation *(P> 0.05)* in the appearance, EE%, VS, PDI, ZP, and RE% of F1 at the end of the storage period. These findings suggested that F1 was stable under the assigned conditions.

#### Determination of F1 cytotoxicity (IC_50_) on liver carcinoma (Hep-G2) cell line

The results described in (Table S2) show that the incubation of the optimum formula (F1) at different concentrations of CBE (31.25, 62.50, 125, 250, 500, and 1000 µg/ml) with Hep-G2 liver carcinoma cell line resulted in viability (%) of 99.06, 98.97, 50.32, 24.36, 18.39 and 10.92, respectively, and toxicity (%) of 0.93, 1.02, 49.67, 75.6, 81.06 and 89.07, respectively. Therefore, the IC_50_ value of F1 against Hep-G2 cells is equal to 166.78 µg/ml.

F1 has been shown to have cytotoxic and apoptotic effects on the liver carcinoma cell line (Hep-G2). This may be attributed to the presence of flavonols, anthocyanins, proanthocyanidins, catechins, phenolic acids, and triterpenoids in CBE (Russo et al., 2020). Polyphenols can prevent cancer initiation and promotion through various mechanisms, including inhibition of the activation of oncogenes and genes involved in oxidative stress and inflammation (Bujak et al., 2021). Recent studies have shown polyphenols to protect against carcinogenesis by modulating epigenetic aberrations such as histone modifications, DNA methylation, and microRNAs (Trautwein et al., 2015).

### In-vivo studies of the optimum CBE loaded BSALs

#### Determination of LD_50_ of F1

The results reported in (Table S3) show that the oral administration of F1 in doses of 350, 500, 800, 1200, 1600, and 1800 mg/kg resulted in mortalities of 0, 1, 3, 6, 9, and 10 rats, respectively. The dose of F1 that resulted in 50% mortality in rats (LD_50_) was found to be 1062.5 mg/kg.

#### Induction of liver injury with DMN

One of the most thoroughly investigated experimental models for elucidating the hepatic injury mechanism is DMN-induced hepatotoxicity in rats. DMN is reported to produce reactive oxygen species (ROS) on its own, leading to a surge in liver enzymes and lipid peroxidation, as well as a considerable elevation in MDA, and a drop in antioxidant enzyme activity in the liver tissues (Lin et al., 2012). Moreover, ROS induces liver fibrosis, cholestasis, hepatic inflammation, and necrosis of liver cells (Tilg & Diehl 2000). DMN intoxication damaged liver function, activated transforming growth factor beta 1 signaling, and increased the expression of α-smooth muscle actin (α-SMA), matrix metalloproteinase-2, and collagen (Lee et al., 2013). Hence, this method is appropriate for determining the efficiency and hepatoprotective capability of natural and synthetic compounds (Ahmad et al., 2014). Therefore, this study was undertaken to assess the hepatoprotective potential of CBE solution and the optimum CBE loaded BSALs (F1).

#### Determination of liver function biomarkers

**Figure 4.**
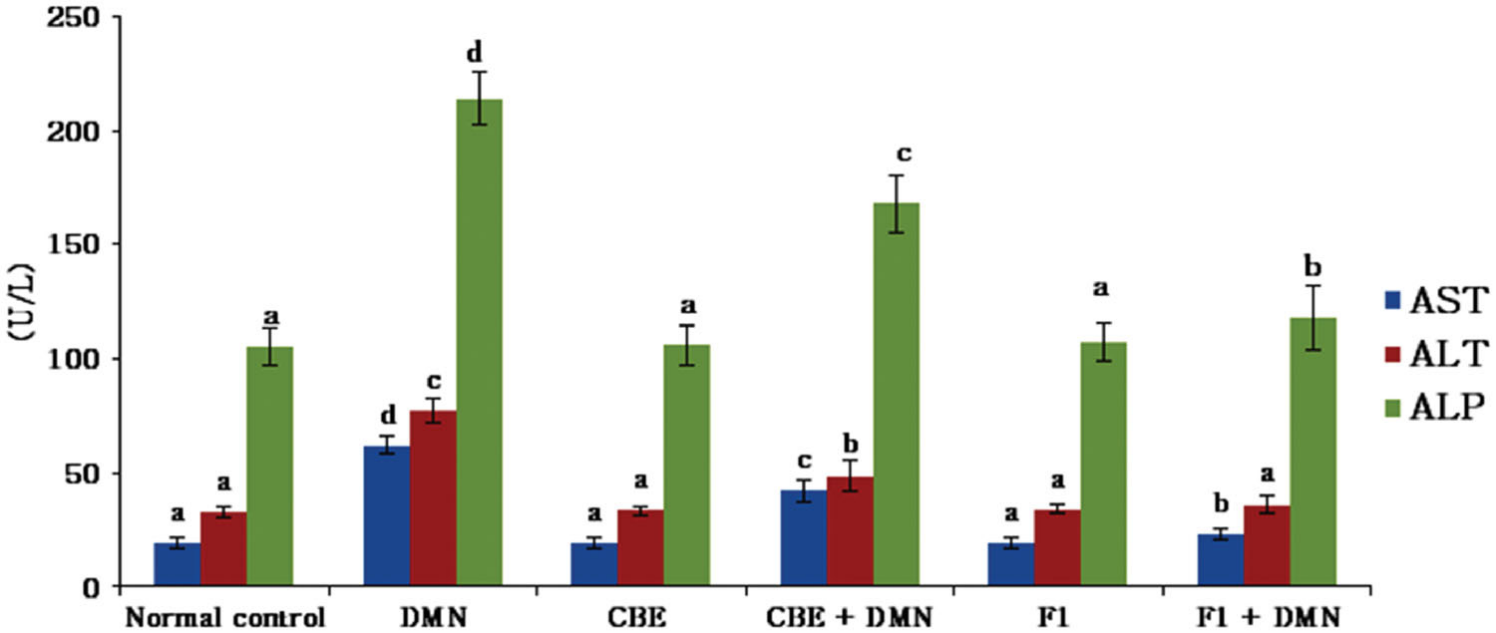
Changes in AST, ALT, ALP serum levels in different groups of rats. Values are represented as mean ± SD (*n* = 10). Similar symbols within the same parameter mean non-significant while different symbols mean significant at *p* < .05 (Duncan’s test).

Hepatotoxicity is manifested by significant elevations in serum AST, ALT, and ALP levels indicating the severity of the hepatic injury (Sallie et al., 1991). Figure 4 summarizes the effect of CBE and the optimum CBE loaded BSALs (F1) on these liver function biomarkers.

In comparison to the normal control group (I), serum AST, ALT, and ALP levels were significantly elevated in the DMN-intoxicated group (II) by 227.5%, 137.33%, and 103.41%, respectively, (*p* < .01), indicating an chronic liver injury. CBE + DMN treated rats (group IV) exhibited significantly reduced serum levels of AST, ALT, and ALP by 32.05%, 37.17%, and 21.55%, respectively, in comparison to the DMN treated group, (*p* < .01). Similarly, a significant reduction in AST, ALT, and ALP serum levels by 62.62%, 53.41%, and 44.78%, respectively, was noticed in F1 + DMN treated rats (group VI) compared to the DMN treated group (II), (*p* < .01). On the other side, non-significant changes were found in the level of these parameters between groups I, III, and V. The aforementioned results confirmed that CBE loaded BSAL (F1) has a higher potential effect in protecting the liver cells against injury compared to CBE solution.

#### Measurement of oxidative stress (homogenate redox) biomarkers

Exposure to DMN causes the production of ROS, which results in an increase in MDA, the end product of lipid peroxidation, and a decrease in SOD, an antioxidative enzyme, Gpx, and GSH (Hong et al., 2011).

**Figure 5.**
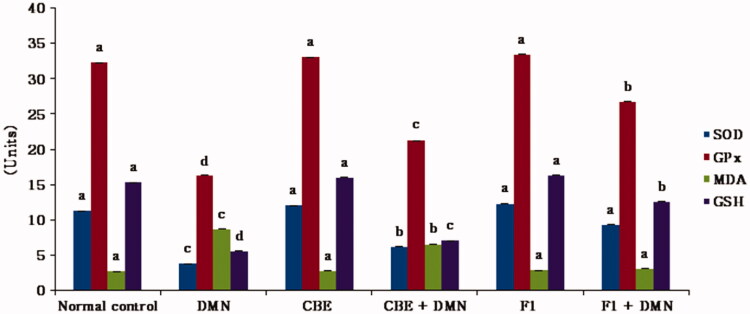
Changes in liver GSH, MDA levels and the activities of SOD and GPx of rats in different groups. Values are represented as mean ± SD (*n* = 10). Similar symbols within the same parameter mean non-significant while different symbols mean significant at *p* < .05 (Duncan’s test).

The present study focuses on the measurement of the activities of the aforementioned biomarkers to demonstrate the extent of the oxidative damage in the liver of animals exposed to DMN and the hepatoprotective effect acquired following the administration of CBE and CBE loaded BSAL (F1), as presented in (Figure 5).

A significant increase in the MDA formation (220.74%) was detected obviously in DMN group (II) animals in contrast to the normal group (I), (*p* < .01). However, rats treated with DMN had a significant decrease in liver SOD, GPx, and GSH levels (*p* < .01), by 66.54%, 49.47%, and 63.58%, respectively, compared to the normal control group.

Regarding group IV (CBE + DMN), the administration of CBE resulted in a significant decrease in MDA formation by 24.48% compared to the DMN group (II), (*p* < .01). In addition, a significant elevation in liver SOD, GPx, and GSH levels by 63.93%, 30.36%, and 39.98% was observed, respectively, compared with the DMN treated group of rats (*p* < .01).

It was evident that there is a significant decrease in MDA formation by 64.66% in (F1 + DMN) group (VI) in comparison to the DMN treated group (*p* < .05). Additionally, SOD, GPx, and GSH levels increased significantly by 121.75%, 32.88%, and 53.59%, respectively, compared to the DMN-treated group of rats (*p* < .05). Meanwhile, non-significant disparities were observed in either the MDA, SOD, GPx, or GSH levels between the normal, CBE and F1 treated animal groups.

The present study demonstrated that CBE loaded BSAL (F1) had attenuated DMN-induced hepatic injury, as evidenced by the significant decrease in serum level of MDA, and elevation of GSH, as well as SOD and GPX activities, confirming its hepatoprotective effect through prevention of membrane damage and their superiority over CBE solution.

#### Measurement of inflammatory and proinflammatory factors

**Figure 6.**
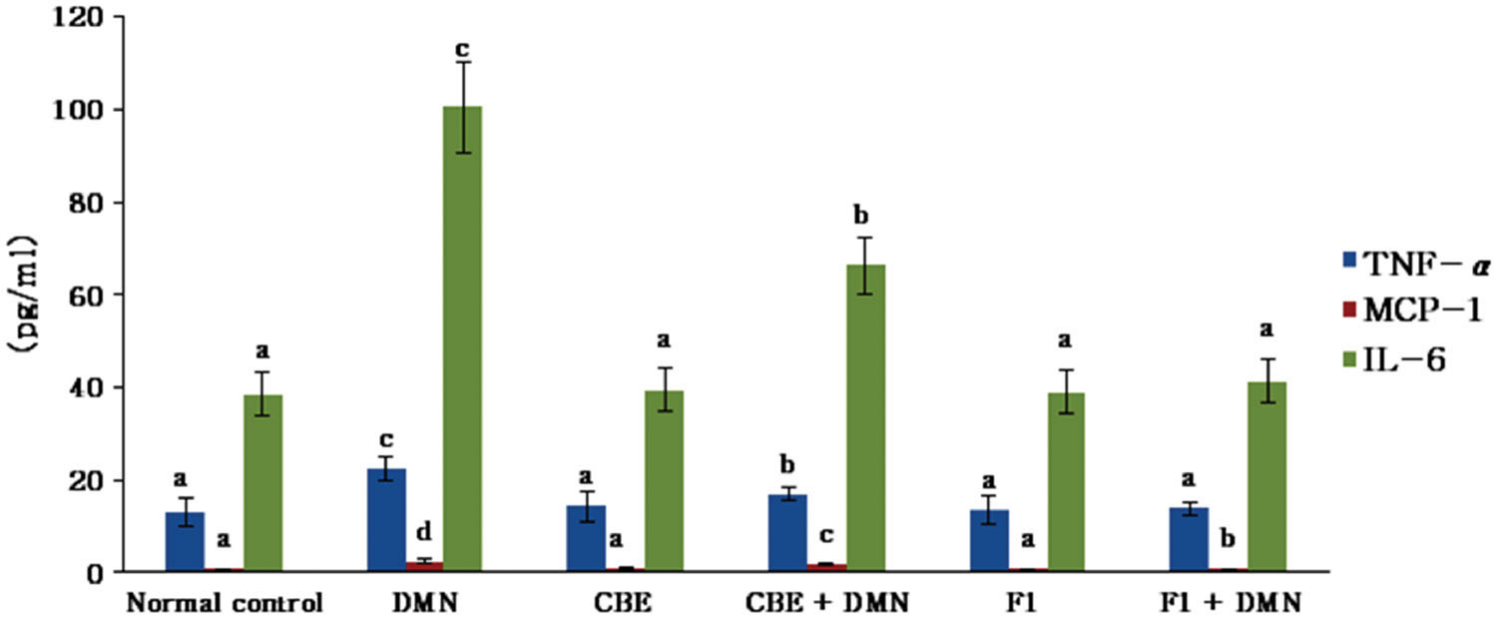
Changes in liver TNF-α, MCP-1 and IL-6 of rats in different groups. Values are represented as mean ± SD (*n* = 10). Similar symbols within the same parameter mean non-significant while different symbols mean significant at *p* < .05 (Duncan’s test).

ROS, as cytotoxic and signaling agents, perform a crucial role in the pathogenesis of inflammatory liver damage (Jaeschke, 2000). It is a fact that hepatocyte injury is followed by an inflammatory response and an elevation in cytokines as TNF-α, MCP-1, and IL-6 which cause uncontrolled damaging effects. Therefore, many investigations have focused on their harmful actions (Anderson & Borlak, 2008). Figure 6 depicts the effect of CBE and the optimum CBE loaded BSALs (F1) on serum TNF-α, IL-6, and MCP-1 levels.

Serum TNF-α, IL-6, and MCP-1 levels in the DMN group (II) were significantly increased by 69.57%, 161.80%, and 235.61%, respectively, when compared to the normal control group (I), (*p* < .01). CBE significantly lowered the levels of liver TNF-, MCP-1, and IL-6 in the CBE + DMN treated group (IV) by 23.89%, 27.76%, and 33.91%, respectively (*p* < .01), when compared to the DMN group (II). However, when rats in group VI (F1 + DMN) were treated with F1, the levels of liver TNF-, MCP-1, and IL-6 were reduced by 38.03%, 66.12%, and 58.92%, respectively (*p* < .01), compared to the DMN group. Alternatively, non-significant changes were noted in the level of these parameters between the normal, CBE, and F1 animals.

These results exposed a remarkable hepatoprotective efficacy of F1 compared to CBE solution and, thus, confirmed the capacity of the BSALs to augment cellular uptake.

### RNA *extraction and real-time polymerase chain reaction (RT-PCR)*

STAT3 is involved and activated in a wide range of tumors, including hepatocellular carcinoma (HCC) of various tissues and organs. Besides the well-established noxae that may be the causative factors for HCC development (Whittaker et al., 2010), the inflammatory conditions have been shown to promote hepatocyte cycling and carcinogenesis, in part through paracrine IL-6 release by macrophages, which results in the activation of STAT3 in hepatocytes. Accordingly, IL-6-deficiency inhibited Diethylnitrosamine (DEN)-initiated proliferation and HCC development, which was linked with reduced STAT3 activation in hepatocytes (Naugler et al., 2007). Additionally, it has been demonstrated that IL-6 and tumor necrosis factor alpha (TNF-γ) are the key factors in liver tumorigenesis in genetically modified mice (Park et al., 2010).

Furthermore, IFN-γ is involved in a variety of inflammatory diseases (Ishida et al., 2004). STAT3 has recently been demonstrated to promote the vascular endothelial growth factor (VEGF), which aids in tumor-associated angiogenesis (Lee et al., 2015). Besides, STAT3 inhibits the proinflammatory cytokines and chemokines in tumor expression, leading to decreased antitumor immune activation (Aguilar-Cazares et al., 2019).

**Figure 7.**
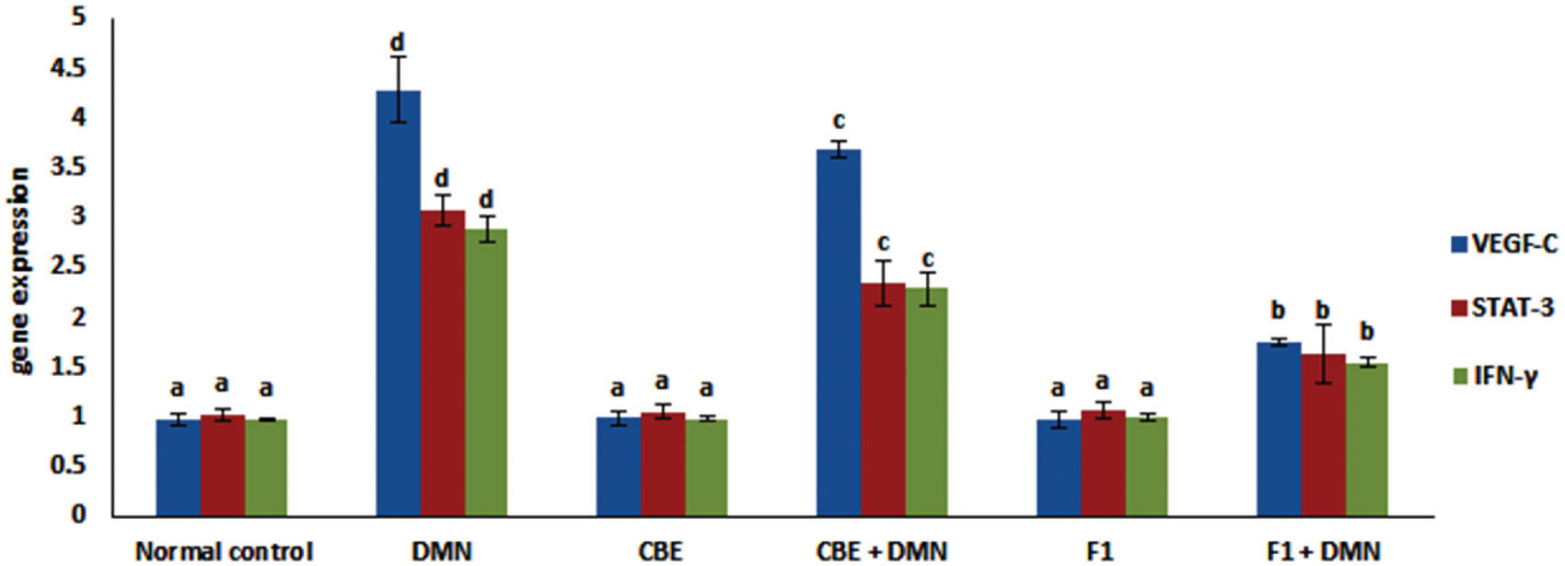
Changes in mRNA levels of VEGF-C, STAT-3 and (IFN)-γ in the liver of rats in different groups. Values are represented as mean ± SD (*n* = 10). Similar symbols within the same parameter mean non-significant while different symbols mean significant at *p* < .05 (Duncan’s test).

Figure 7 demonstrates the effect of CBE and the optimum CBE loaded BSALs (F1) on mRNA expression level of STAT-3, IFN-γ and VEGF-C. Compared to the normal control group (I), gene expression of VEGF-C, STAT-3, and IFN-γ was upregulated by 392.26%, 199.03%, and 196.90%, respectively, in the DMN-intoxicated group (II). When rats were given CBE + DMN (IV), their liver VEGF-C, STAT-3, and (IFN)-γ gene expression decreased by 14.21%, 24.03%, and 20.48%, respectively, in comparison to the DMN-treated group (II) (*p* < .05).

Additionally, F1 + DMN administration (group VI) resulted in a significant decrease in liver VEGF-C, STAT-3, and (IFN)-γ gene expression by 59.21%, 46.8%, and 53.63%, respectively, compared to the DMN-treated group (II) of rats (*p* < .05). Quantitative real-time PCR analysis disclosed that levels of VEGF-C, STAT-3 and IFN-γ mRNA expression in the liver of normal animals that received either CBE or F1 was not significantly altered compared to the normal control group (*p* > .05).

Therefore, the pronounced ability of CBE loaded BSAL (F1) to decrease the previously mentioned liver gene expression significantly established its potential hepatoprotective activity.

In a nutshell, CBE is rich in flavonoids and polyphenols, which play a potent antioxidant role by scavenging the ROS induced by DMN, thereby decreasing the process of lipid peroxidation and protecting the liver against damage. In comparison to CBE solution, our study confirmed the boosting of the potential hepatoprotective effect of CBE when it was incorporated into liposomes augmented with SGC. This may be attributed to the high permeability and stability in the gastrointestinal tract achieved by the presence of SGC (Niu et al., 2012), as well as the hepatoprotective effect of phosphatidylcholine and its inherent ability to restore intestinal barrier function and diminish endotoxemia (Chen et al., 2019).

## Conclusion

CBE loaded BSALs were fabricated using the thin film hydration technique, applying 2^3^ full factorial design to choose the optimum formulation. F1 exhibited a high EE%, charged vesicle with a small size, and a sustained release pattern. TEM revealed spherical, non-aggregated vesicles. Moreover, in-vivo studies demonstrated the superiority of F1 compared to CBE solution in enhancing the hepatoprotective activity by raising the levels of the antioxidant enzymes and decreasing the levels of inflammatory mediators in rats with DMN-induced hepatic injury. Thereby, it is possible to conclude that CBE loaded BSALs might be effectively utilized in hindering liver damage in the future.

## Supplementary Material

Supplemental Material
