# Supplementary material for: Design and optimization of cranberry extract loaded bile salt augmented liposomes for targeting of MCP-1/STAT3/VEGF signaling pathway in DMN-intoxicated liver in rats
Source: Drug Deliv. 2022 Jan 31;29(1):427–39. doi: 10.1080/10717544.2022.2032875 (PMC8812757; doi:10.1080/10717544.2022.2032875)
Supplement: Supplemental Material [file IDRD_A_2032875_SM8961.docx]

Design and optimization of cranberry extract loaded bile salt augmented liposomes for targeting of MCP-1/STAT3/VEGF signaling pathway in DMN‑intoxicated liver in rats

Sara M Soliman^a,^, Shaimaa Mosallam^a^, Mohamed A. Mamdouh^a^, Mohammed Abdalla Hussein^b^, Shady M. Abd El-Halim^a^

*^a^Department of Pharmaceutics and Industrial Pharmacy, Faculty of Pharmacy, October 6 University, 6^th^ of October City, Giza 12585, Egypt*

*^b^Biotechnology Department, Faculty of Applied Health Sciences Technology, October 6 University, 6^th^ of October City, Giza 12585, Egypt*

**Corresponding Author**: Sara M. Soliman.

**Postal address**: Department of Pharmaceutics and industrial pharmacy, Faculty of Pharmacy, October 6 University, Central Axis, 6^th^ of October city, Giza, Egypt.

**Postal** **code**: 12585

**E-mail address**: [sara.soliman@o6u.edu.eg](mailto:sara.soliman@o6u.edu.eg)

[sara_dyaa2005@yahoo.com](mailto:sara_dyaa2005@yahoo.com)

**Cell phone number**: 00201001421958

Materials

Cranberry Extract (CBE), methylene chloride, methanol and dimethyl nitrosamine (DMN) were purchased from Sigma Aldrich Chemical Co., St Louis, Missouri, USA. Sodium glycocholate (SGC) was kindly obtained from BASF Co., NJ, USA. Epikuron 100 (soyabean lecithin containing 20% Phosphatidylcholine) and Epikuron 200 (soyabean lecithin containing 92% Phosphatidylcholine) were kindly provided by Cargill, Deutschland GmbH & Co., Hamburg, Germany. All other chemicals used were of analytical grade.

**Instruments**

Rotary-evaporator (Rotavapor, Heidolph VV 2000, Burladingen, Germany). Probe sonication with an Ultrasonic Processor (Chrom Tech, model UP-500, England). Centrifugal filter units (Amicon® Ultra-4, 3K, Millipore, Germany). Cooling centrifuge (Sigma 3-30 KS, Germany). UV/VIS spectrophotometer (Shimadzu, model UV-1601 PC, Kyoto, Japan). Malvern Zetasizer (Malvern Instrument Ltd., Worcestershire, UK). Cellulose dialysis membrane with molecular weight cutoff 12.000-14,000 Da (Spectrum Laboratories Inc., Rancho Dominguez, CA). Hotplate magnetic stirrer (7280; Ugo Basile, Italy). Transmission Electron Microscopy (TEM, JEOL, JEM-1230, Japan).

**Table S1.** The Primer sequences of STAT-3, IFN-γ, VEGF-C and GAPDH.

| **Gene** | **Primer sequence** |
| --- | --- |
| **STAT-3** | F: 5′-GACCCGCCAACAAATTAAGA-3′  R: 5′-TCGTGGTAAACTGGACACCA-3′ |
| **IFN-γ** | F:5′-AAAGACAACCAGGCCATCAGCAAC-3′  R: 5′-TCTGTGGGTTGTTCACCTCGAACT-3′ |
| **VEGF-C** | F: 5′-AACGTGTCCAAGAAATCAGCC-3′  R: 5′-AGTCCTCTCCCGCAGTAATCC-3′. |
| **GAPDH**  **(internal control for qRT-PCR)** | F: 5′-CTCAACTACATGGTCTACATGTTCCA-3′  R: 5′-CCATTCTCGGCCTTGA-CTGT-3’. |

**Table S2.** IC_50_ of the optimum CBE loaded BSALs (F1) against liver carcinoma (Hep-G2) cell line.

| **Conc. ug/ml** | **O.D** | | | **Mean O.D** | **SE** | **Viability %** | **Toxicity %** |
| --- | --- | --- | --- | --- | --- | --- | --- |
| **DMSO (0.1%) 1:2** | 0.374 | 0.342 | 0.355 | 0.357 | 0.009292 | 100 | 0 |
| **1000** | 0.032 | 0.041 | 0.044 | 0.039 | 0.003606 | 10.92436975 | 89.07563025 |
| **500** | 0.065 | 0.074 | 0.058 | 0.065667 | 0.004631 | 18.39402428 | 81.60597572 |
| **250** | 0.086 | 0.092 | 0.083 | 0.087 | 0.002646 | 24.3697479 | 75.6302521 |
| **125** | 0.169 | 0.198 | 0.172 | 0.179667 | 0.009207 | 50.32679739 | 49.67320261 |
| **62.5** | 0.363 | 0.342 | 0.355 | 0.353333 | 0.006119 | 98.9729225 | 1.027077498 |
| **31.25** | 0.359 | 0.36 | 0.342 | 0.353667 | 0.00584 | 99.06629318 | 0.933706816 |
| **IC_50_** | **166.78** |  |  |  |  |  |  |

Conc: Concentration; O.D: Optical density; SE: Standard Error.

**Table S3.** Determination of LD_50_ of the optimum CBE loaded BSALs (F1).

| **Groups** | **Dose (mg/kg)** | **No. of dead animals** | **(Z)** | **(d)** | **(Z.d)** |
| --- | --- | --- | --- | --- | --- |
| **1** | 350 | 0 | 0.5 | 150 | 75 |
| **2** | 500 | 1 | 2.0 | 300 | 600 |
| **3** | 800 | 3 | 4.5 | 400 | 1800 |
| **4** | 1200 | 6 | 7.5 | 400 | 3000 |
| **5** | 1600 | 9 | 9.5 | 200 | 1900 |
| 6 | 1800 | 10 | ---- | ---- | ---- |
| ∑ (Z.d) | | | | | 7375 |

Total number of animals used in each group (n) = 10; Z: mean of dead animals between two successive groups; d: the interval between each two successive doses; ∑ (Z.d): the sum of (Z x d).
